# Deferasirox treatment of iron-overloaded chelation-naïve and prechelated patients with myelodysplastic syndromes in medical practice: results from the observational studies eXtend and eXjange

**DOI:** 10.1111/j.1600-0609.2011.01726.x

**Published:** 2012-03

**Authors:** Norbert Gattermann, Andrea Jarisch, Rudolf Schlag, Klaus Blumenstengel, Mariele Goebeler, Matthias Groschek, Christoph Losem, Maria Procaccianti, Alexia Junkes, Oliver Leismann, Ulrich Germing

**Affiliations:** 1 Department of Hematology, Oncology and Clinical Immunology, Heinrich-Heine-UniversityDüsseldorf; 2 Johann Wolfgang Goethe-University, Hospital for Children and AdolescentsFrankfurt am Main; 3 Hematology and Oncology PracticeWürzburg; 4 Internal Medicine, Hematology and Oncology PracticeEisenach; 5 Department of Hematology and Oncology, University Hospital WürzburgWürzburg; 6 Internal Medicine, Hematology and Internal Oncology PracticeWürselen; 7 Hematology and Oncology PracticeNeuss; 8 Hematology, Oncology and Infection PracticeKarlsruhe; 9 Business Unit Oncology, Novartis Pharma GmbHNuremberg, Germany

**Keywords:** deferasirox, oral, myelodysplastic syndromes, iron overload, iron chelation, serum ferritin, safety

## Abstract

EXtend and eXjange were prospective, 1-yr, non-interventional, observational, multicentre studies that investigated deferasirox, a once-daily oral iron chelator, in iron-overloaded chelation-naïve and prechelated patients with myelodysplastic syndromes (MDS), respectively, treated in the daily-routine setting of office-based physicians. No inclusion or exclusion criteria or additional monitoring procedures were applied. Deferasirox was administered as recommended in the European Summary of Product Characteristics. Haematological parameters and adverse events (AEs) were collected at two-monthly intervals. Data from 123 chelation-naïve patients with MDS (mean age 70.4 yrs) with median baseline serum ferritin level of 2679 (range 184–16 500) ng/mL, and 44 prechelated patients with MDS (mean age 69.6 yrs) with median baseline serum ferritin level of 2442 (range 521–8565) ng/mL, were assessed. The mean prescribed daily dose of deferasirox at the first visit was 15.7 and 18.7 mg/kg/d, respectively. Treatment with deferasirox produced a significant reduction in median serum ferritin levels in chelation-naïve patients with MDS from 2679 to 2000 ng/mL (*P* = 0.0002) and a pronounced decrease in prechelated patients with MDS from 2442 to 2077 ng/mL (*P* = 0.06). The most common drug-related AEs were gastrointestinal, increased serum creatinine levels and rash. These studies demonstrate that deferasirox used in physicians’ medical practices is effective in managing iron burden in transfusion-dependent patients with MDS.

With the availability of agents such as lenalidomide and hypomethylating agents, the proportion of patients with myelodysplastic syndromes (MDS) that are successfully managed has improved in recent years (1). Nonetheless, supportive care with red blood cell (RBC) transfusions remains the mainstay of treatment (2, 3). The majority of patients with MDS are chronically transfused as a result of anaemia and can therefore develop a high iron load. This can be exacerbated via increased dietary iron absorption as a result of ineffective erythropoiesis (4, 5). Iron overload is known to lead to progressive damage in organs such as the liver, heart and endocrine glands. Those who become transfusion dependent can develop substantial morbidity and significantly reduced overall and leukaemia-free survival (6–9).

Although the evidence for the benefits of iron chelation therapy to treat iron overload in patients with thalassaemia is unequivocal and well founded, its use in patients with MDS remains controversial because of a lack of definitive evidence documenting its benefits (10). Nonetheless, current guidelines on the treatment of transfusion-dependent patients with MDS recommend the use of iron chelation therapy in iron-overloaded patients (1, 11–13). Furthermore, several recent studies have demonstrated that iron chelation significantly improves survival in heavily transfused patients with MDS (14–16). One study demonstrated that the median overall survival from diagnosis was 124 months in chelated and 53 months in non-chelated patients (*P* < 0.0003), even after adjustments were made for other prognostic factors such as age and transfusion requirement (16).

Deferasirox, a once-daily oral iron chelator, has been shown to maintain or reduce body iron in patients with MDS [assessed by liver iron concentration (LIC) and serum ferritin concentrations] in a number of studies (17–19). In the largest of these, enrolling 341 patients with MDS, deferasirox markedly reduced median serum ferritin in both chelation-naïve patients and those who had previously received iron chelation therapy (19). However, despite 5 yrs of clinical experience with deferasirox in the public domain, there is limited evidence regarding the effects of iron chelation with deferasirox in iron-overloaded patients with MDS treated in clinical practice.

Here, we report results from the eXtend and eXjange studies, which investigated deferasirox therapy in chelation-naïve and prechelated patients, respectively, outside the setting of a controlled clinical trial. The objective of the two studies was to evaluate the efficacy, safety and tolerability of deferasirox for the treatment of transfusion-dependent, iron-overloaded patients in a daily-routine situation of office-based physicians. The trials enrolled patients with a range of anaemias; however, this report will focus on data from the patients with MDS while presenting only key data from patients with other disease types.

## Methods

### Study designs

Both the eXtend and eXjange studies were prospective, 1-yr, non-interventional, observational, open-label multicentre studies conducted in Germany. The studies enrolled iron-overloaded male and female patients with MDS and other transfusion-dependent anaemias. Patients were eligible for deferasirox therapy as stated in the European Summary of Product Characteristics (SPC) and the terms of the Exjade marketing authorization in Germany (Fachinformation) (20). Iron overload in the studies was defined as per the SPC (serum ferritin > 1000 ng/mL or approximately 20 units of packed RBCs). No further inclusion or exclusion criteria were applied in addition to the contraindications noted in the SPC. In the analysis presented here, data from patients with inherited haemoglobinopathies (including β-thalassaemia, sickle cell disease, α-thalassaemia, congenital aplastic anaemia, hereditary haemolytic anaemia and haemochromatosis) were not included.

Because deferasirox was administered in the attending physicians’ regular medical practices, no additional diagnostic or monitoring procedures were utilised and no interventions in therapeutic decisions made by the investigators were allowed; therefore, approval from ethics committees was not required.

### Deferasirox dosing

As recommended in the SPC, deferasirox in both studies was prescribed at a starting dose of 20 mg/kg/d, with the exception of patients who required a reduction in elevated body iron levels and who were receiving >14 mL/kg/month of packed RBCs; in these patients, the initial deferasirox dose was 30 mg/kg/d. In patients who did not require a reduction in body iron levels and who were receiving <7 mL/kg/month of packed RBCs, the initial deferasirox dose was 10 mg/kg/d. In those who were prechelated and well managed on deferoxamine, the initial deferasirox dose was numerically half that of their deferoxamine dose.

Dose adjustments were allowed in both studies and implemented according to instructions in the SPC. Doses could be increased or decreased every 3–6 months in steps of 5–10 mg/kg/d, in response to trends in serum ferritin levels and according to the individual patient’s therapeutic goals (maintenance or reduction of iron burden).

### Assessments

Patients had an initial visit with their physician at baseline and follow-up visits at approximately 2, 4, 6, 8, 10 and 12 months. At each visit, recorded data included laboratory examinations (e.g. serum ferritin and serum creatinine levels and creatinine clearance), number of blood transfusions received, daily deferasirox dose and any change in dose, as well as changes in concomitant medications. As a result of the non-interventional study design reflecting regular daily medical practices, a central laboratory was not used for analyses. Any premature discontinuation of therapy or occurrences of adverse events (AEs) were also reported. The final visit was defined as the last available follow-up.

### Statistical analysis

All patients who took at least one dose of deferasirox and had any follow-up information after their initial visit were included in the intent-to-treat analysis. Descriptive analyses of the data were performed using summary statistics for categorical and quantitative data. Continuous data were described by mean, standard deviation (SD) or median (range). The non-parametric Wilcoxon signed-rank test was used to calculate *P*-values for changes in serum ferritin levels. For comparison of the number of incidences of AEs leading to discontinuations, Fisher’s exact test was used. Statistics were calculated in an exploratory manner.

## Results

### Patient population

Between October 2006 and January 2009, 335 chelation-naïve and prechelated patients were included in the two trials from 76 and 37 participating physicians in Germany, respectively (Table 1). A total of 167 (49.9%) patients were diagnosed with MDS, 123 in the eXtend study and 44 in the eXjange study (Table 2).

**Table 1:** Baseline characteristics

	Chelation-naïve (eXtend)			Prior chelation (eXjange)		
		
Characteristics	MDS (*n* = 123)	Other^1^ (*n* = 91)	All patients (*n* = 214)	MDS (*n* = 44)	Other^2^ (*n* = 25)	All patients (*n* = 69)
Mean age ± SD, yrs	70.4 ± 10.7	66.1 ± 13.0	68.6 ± 11.9	69.6 ± 8.4	57.2 ± 21.6	65.2 ± 15.5
Female : male : missing, *n*	58 : 65 : 0	40 : 49 : 2	98 : 114 : 2	20 : 24 : 0	12 : 13 : 0	32 : 37 : 0
Race, *n* (%)						
Caucasian	110 (89.4)	76 (83.5)	186 (86.9)	39 (88.6)	24 (96.0)	63 (91.3)
Asian	0	1 (1.1)	1 (0.5)	0	0	0
Other	7 (5.7)	5 (5.5)	12 (5.6)	3 (6.8)	0	3 (4.3)
Missing	6 (4.9)	9 (9.9)	15 (7.0)	2 (4.5)	1 (4.0)	3 (4.3)
Prior chelation therapy, *n* (%)						
Deferoxamine	–	–	–	37 (84.1)	23 (92.0)	60 (87.0)
Deferiprone	–	–	–	10 (22.7)	9 (36.0)	19 (27.5)
Other or unknown	–	–	–	3 (6.8)	1 (4.0)	4 (5.8)
Median duration of prior chelation (range), yrs						
Deferoxamine	–	–	–	2.0 (0.3–16.4)	1.5 (0.3–6.3)	1.6 (0.3–16.4)
Deferiprone	–	–	–	1.3 (0.8–3.8)	2.0 (0.3–2.7)	1.8 (0.3–3.8)
Median transfused blood volume per month (range), mL	1000 (0–4000)	660 (0–4200)	990 (0–4200)	1000 (167–3960)	870 (0–2640)	1000 (0–3960)
Mean iron intake ± SD, mg/kg/d	0.36 ± 0.27	0.30 ± 0.24	0.33 ± 0.26	0.44 ± 0.53	0.28 ± 0.30	0.39 ± 0.47
Median serum ferritin (range), ng/mL	2679 (184–16 500)	2940 (763–11 423)	2889 (184–16 500)	2442 (521–8565)	2232 (539–9427)	2378 (521–9427)

MDS, myelodysplastic syndromes.

1 Most common other anaemias in eXtend included myelofibrosis (*n* = 26), myeloproliferative disorder (*n* = 11) and acute myeloid leukaemia (*n* = 11).

2 Most common other anaemias in eXjange included myelofibrosis (*n* = 6), myeloproliferative disorder (*n* = 4) and acute myeloid leukaemia (*n* = 4).

**Table 2:** International Prognostic Scoring System (IPSS), WHO and karyotype classification of patients with MDS

Characteristics	Chelation-naïve (*n* = 123)	Prior chelation (*n* = 44)
IPSS, *n* (%)		
Low	26 (21.1)	14 (31.8)
Intermediate-1	39 (31.7)	16 (13.0)
Intermediate-2	14 (11.4)	2 (4.5)
High	4 (3.3)	0
Missing	40 (32.5)	12 (27.3)
Karyotype, *n* (%)		
Good	45 (36.6)	23 (52.3)
Intermediate	24 (19.5)	2 (4.5)
Poor	4 (3.3)	0
Missing	50 (40.7)	19 (43.2)
WHO, *n* (%)		
RA	22 (17.9)	17 (38.6)
RARS	30 (24.4)	10 (22.7)
RCMD	10 (8.1)	2 (4.5)
RCMD-RS	0	2 (4.5)
RAEB-1	12 (9.8)	2 (4.5)
RAEB-2	8 (6.5)	1 (2.3)
MDS-U	5 (4.1)	1 (2.3)
Del (5q) syndrome	13 (10.6)	4 (9.1)
Missing	23 (18.7)	5 (11.4)

WHO, World Health Organization; RA, refractory anaemia; RARS, RA with ringed sideroblasts; RCMD, refractory cytopenia with multilineage dysplasia; RCMD-RS, RCMD and ringed sideroblasts; RAEB, refractory anaemia with excess blasts; MDS-U, unclassified myelodysplastic syndromes.

The median period of observation of the chelation-naïve and prechelated patients with MDS was 364 d (range 13–511) and 341 d (range 43–606), respectively. At the final visit, 76 (62.3%) and 31 (70.5%) chelation-naïve and prechelated patients, respectively, were continuing to receive treatment. The proportion of patients with MDS receiving treatment at the final visit was slightly higher compared with that observed in chelation-naïve and prechelated patients with other non-hereditary anaemias (*n* = 48; 52.7% and *n* = 13; 52.0%, respectively). Reasons for the discontinuation of deferasirox by the final visit included AEs in 15 (34.1%) and 8 (61.5%) chelation-naïve and prechelated patients with MDS, respectively (*P* = 0.4564). Other reasons for discontinuation included poor compliance in 3 (6.8%) and 1 (7.7%), and other/missing data in 29 (65.9%) and 4 (30.8%) chelation-naïve and prechelated patients, respectively.

Among all patients enrolled, concomitant medications were taken by 137 (60.6%) and 73 (67.0%) patients in the chelation-naïve and prechelated groups, respectively. In both studies, the most frequently taken were antihypertensive, thyroid and antidiabetic medications.

### Blood transfusions during the studies

The mean ± SD number of transfusions received by chelation-naïve and prechelated patients with MDS over the course of both studies was 23.3 ± 16.6 and 26.2 ± 18.7, respectively. This was similar to chelation-naïve patients with other anaemias (20.6 ± 17.4); however, patients with other anaemias who were prechelated had received fewer transfusions (14.2 ± 14.5).

Over the year of observation in both studies, there was no change in patients’ median monthly transfused blood volume.

### Deferasirox dosing

All patients with MDS received deferasirox for similar durations while on the study (Table 3). Chelation-naïve and prechelated patients with other anaemias received deferasirox for a median of 320 d (3–486) and 343 d (10–405), respectively.

**Table 3:** Deferasirox dosing in patients with myelodysplastic syndromes

Dosing	Chelation-naïve (*n* = 123)	Prior chelation (*n* = 44)
Mean starting dose, mg/kg/d	15.7	18.7
Patients starting on 20 to <30 mg/kg/d, *n* (%)	59 (48.3)	22 (50.0)
Median daily deferasirox dose over the course of the studies, mg/kg/d (range)	20 (2.0–30.3)	20 (4.7–35.0)
Median time on deferasirox treatment, d (range)	357 (2–511)	317 (16–463)
Dose adjustments for all starting doses^1^, *n* (%)		
Unchanged	59 (48.0)	22 (50.0)
Increased	8 (6.5)	1 (2.3)
Reduced	5 (4.1)	5 (11.4)
Missing data	50 (40.7)	16 (36.4)
One or two dose interruptions	14 (11.4)	7 (15.9)

1 Baseline compared with final visit.

The mean starting doses for chelation-naïve and prechelated patients with MDS were 15.7 and 18.7 mg/kg/d, respectively (Table 3). Approximately half of the patients with MDS received a starting dose of 20 to <30 mg/kg/d. Of these patients, approximately half did not require a dose change when comparing their first and final visit doses.

**Fig. 1:**
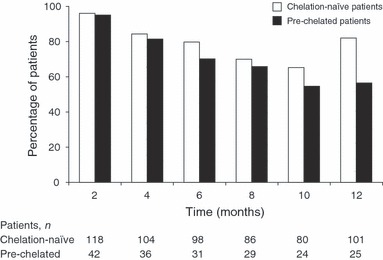
Percentage of chelation-naïve (*n* = 123) and prechelated patients with myelodysplastic syndromes (*n* = 44) who attended follow-up visits.

A large proportion of the patients with MDS attended follow-up sessions, indicating that the number of documented days that these patients were on therapy was high (Fig. 1). The proportion of patients with other anaemias who attended follow-up visits was 92% at 2 months and 68% at 12 months in the chelation-naïve group and 100% at 2 months and 76% at 12 months in the prechelated group.

### Effect of deferasirox on serum ferritin levels

**Fig. 2:**
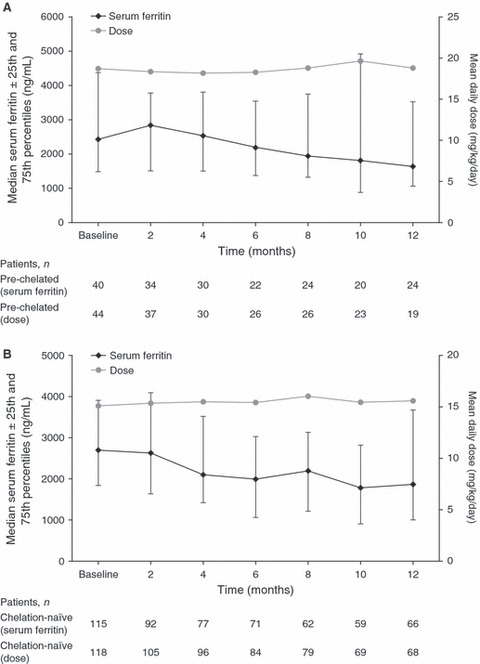
Median serum ferritin levels (±25th and 75th percentiles) and mean daily dose over 12 months in patients with myelodysplastic syndromes who were (A) prechelated and (B) chelation-naïve.

Treatment with deferasirox produced significant reductions in median serum ferritin in chelation-naïve patients with MDS. In those who were prechelated, there was a pronounced but non-significant decrease in median serum ferritin (Table 4 and Fig. 2).

**Table 4:** Changes in median serum ferritin levels in patients with myelodysplastic syndromes

	Serum ferritin levels			
		
Population	Median baseline (range), ng/mL	Median final visit (range), ng/mL	Absolute change, ng/mL^1^	*P-*value^2^
Chelation-naïve (*n* = 123)	2679 (184–16 500)	2000 (155–14 143)	−662	0.0002
Prior chelation (*n* = 44)	2442 (521–8565)	2077 (248–7227)	−716	0.06

1 As the changes in serum ferritin levels were assessed from the individual pre- and post-treatment differences, the medians of these differences do not necessarily correspond to the differences in the medians calculated from the baseline and final visits separately.

2 Based on an exploratory analysis.

In chelation-naïve patients with other anaemias, deferasirox treatment also produced a significant reduction of −783 ng/mL (*P* < 0.0001) in median serum ferritin levels from baseline [2940 ng/mL (763–11 423)] to final visit [2097 ng/mL (109–6899)]; this was similar to that in the chelation-naïve patients with MDS. In prechelated patients with other anaemias, there was a similar reduction (−725 ng/mL) in median serum ferritin from baseline [2232 ng/mL (539–9427)] to final visit [2000 ng/mL (416–5813)], although this was not significant (*P* = 0.2101).

### Safety

Investigator-assessed drug-related AEs were documented in 42 (34.1%) chelation-naïve and 18 (40.9%) prechelated patients with MDS. The majority of these AEs were mild-to-moderate in severity. The most common investigator-assessed drug-related AEs were diarrhoea, nausea, increased serum creatinine levels and rash (Table 5).

**Table 5:** Most common investigator-assessed drug-related adverse events (AEs) (occurring in ≥5% patients with myelodysplastic syndromes)

AEs, *n* (%)	Chelation-naïve (*n* = 123)	Prior chelation (*n* = 44)
Diarrhoea	13 (10.6)	4 (9.1)
Nausea	11 (8.9)	3 (6.8)
Increased serum creatinine	6 (4.9)	4 (9.1)
Rash	8 (6.5)	2 (4.5)

Of the chelation-naïve patients enrolled, the proportion of those with other anaemias with documented investigator-assessed drug-related AEs (*n* = 29; 31.9%) was similar to those with MDS. When comparing to prechelated patients, the proportion of patients with other anaemias and documented investigator-assessed drug-related AEs was higher (*n* = 14; 56.0%), although also most commonly gastrointestinal [nausea (*n* = 4; 16.0%), diarrhoea (*n* = 4; 16.0%)]. In the majority of all chelation-naïve and prechelated patients (60.2% and 71.4%, respectively), the drug-related AEs were resolved.

Serious AEs (SAEs) assessed by investigators as drug related (reported as having a possible or probable cause related to deferasirox) in patients with MDS were reported in 5 (4.1%) chelation-naïve patients and 2 (4.5%) patients who were prechelated. In chelation-naïve patients, these included discoloured faeces, gastrointestinal haemorrhage (one patient each had a colonoscopy and an endoscopy of the upper gastrointestinal tract for the investigation of gastrointestinal haemorrhage), myocardial infarction, neutropenia, cataract, cataract operation complication and renal failure (all *n* = 1). In prechelated patients, these included increased serum creatinine levels, decreased glomerular filtration rate and renal failure (all *n* = 1). Although the myocardial infarction was reported as having a suspected (possible or probable) causality related to deferasirox, this patient had concomitant hypertension and chronic cardiac insufficiency, which may provide a possible explanation for this SAE. Possible drug-related SAEs reported in patients with other anaemias were loss of vision in one chelation-naïve patient with cytochemically differentiated acute myelogenous leukaemia with minimal differentiation (AML-M0) and development of diabetes mellitus in one prechelated patient with large granular leukaemia of T-cell type.

In all patients, six of 10 (60%) drug-related SAEs in the chelation-naïve patients resolved; however, none of four in prechelated patients was resolved (the outcome was reported in two cases). There were a total of 33 (15.4%) and 6 (8.7%) deaths in the eXtend and eXjange studies, respectively, among all patients. In the MDS population, there were a total of 22 deaths, 19 (15.4%) of whom were chelation-naïve and 3 (6.8%) were prechelated. None of the deaths was considered to be related to deferasirox.

**Fig. 3:**
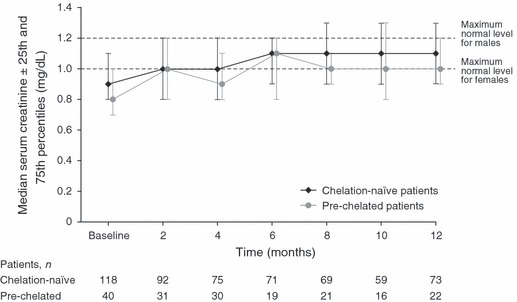
Median serum creatinine levels (±25th and 75th percentiles) over 12 months in patients with myelodysplastic syndromes.

In patients with MDS enrolled across both studies, there were increases in median serum creatinine levels measured as laboratory values from baseline to study end (Fig. 3). Chelation-naïve and prechelated patients had median increases of 0.1 (*P* ≤ 0.0001) and 0.2 mg/dL (*P* = 0.0002), respectively. Fifteen chelation-naïve patients and eight prechelated patients had increases in serum creatinine that were above the maximum normal levels (1.0 mg/dL for women and 1.2 mg/dL for men). Of these patients, 7/15 and 2/8, respectively, had baseline serum creatinine levels that were higher than the maximum normal level.

An increase in alanine transaminase levels was reported as a drug-related AE in 1 (2.3%) prechelated patient with MDS, but in chelation-naïve patients, there were no increases reported as laboratory investigations or AEs.

## Discussion

Deferasirox has been investigated in patients with MDS in a range of clinical studies, demonstrating the efficacy of deferasirox to significantly reduce or maintain serum ferritin levels in transfusion-dependent, iron-overloaded patients (17–19). However, ‘real-world’ studies of iron chelation therapy, particularly deferasirox, in patients with MDS are limited in the published literature (21–23). The eXtend and eXjange studies aimed to contribute to the current understanding of chelation therapy in patients with MDS by presenting additional data on the efficacy, safety and tolerability of deferasirox in a large population of patients with MDS treated for iron overload in the clinical practice setting.

Patients enrolled on these studies had high median baseline serum ferritin levels. In the chelation-naïve patients with MDS, the median baseline serum ferritin level was above the threshold (2500 ng/mL) known to be associated with significant clinical complications (24). This observation indicates that these patients may have benefited from the initiation of iron chelation therapy earlier in the course of their disease. Prechelated patients with MDS had a median baseline serum ferritin level that was near to the threshold, suggesting that the previous management of these patients’ iron load was suboptimal. This may reflect the burdensome infusions required with deferoxamine, and the associated poor compliance (25). It may also be related to the controversy surrounding the use of iron chelation therapy in patients with MDS (10). In a recent observational study conducted in patients with low-risk MDS in Spain (≤65 yrs old with serum ferritin levels >1000 ng/mL), 75% of patients had received >20 RBC units, while only 38% were receiving iron chelation therapy, 92% of whom with deferoxamine. Of those receiving iron chelation, 24% had serum ferritin levels >2500 ng/mL at the start of therapy (22). The authors concluded that despite scientific evidence supporting the use of chelation therapy in transfusion-dependent patients with MDS, chelation is not as widely used as it should be.

Consistent with the use of serum ferritin level measurement as a marker of iron burden in clinical trials and real-life practice (18, 19, 26, 27), serum ferritin level of the patient was also employed as an efficacy endpoint in these observational studies. Selection of serum ferritin as a clinical efficacy measure in this naturalistic setting facilitates comparisons with clinical trial findings and enables the presentation of efficacy data on deferasirox that is more readily applicable to clinical practice. The eXtend study demonstrated that 12 months of deferasirox therapy in the setting of office-based physicians produced a significant decrease in the serum ferritin level of chelation-naïve patients. The decrease was similar to that in chelation-naïve patients with MDS in the recent prospective, open-label EPIC [Evaluation of Patients’ Iron Chelation with Exjade(R)] study, which enrolled 341 patients with MDS (19). The study demonstrated a significant decrease from 2716 to 1754 ng/mL after 1 yr of deferasirox therapy, administered at a mean average actual dose of 19.2 mg/kg/d (19). In both the eXjange and the EPIC studies, prechelated patients with MDS also demonstrated a decrease in serum ferritin levels, although this did not achieve statistical significance in either study. More recently, a prospective, open-label US study (27), although enrolling a smaller number of patients with MDS (*n* = 24) than reported here or in the EPIC study, showed a 39% decrease in serum ferritin levels from 4416 ng/mL at baseline to 2685 ng/mL in patients who completed 1 yr of treatment with deferasirox (*n* = 9, mean deferasirox dose of 20.1 mg/kg/d) and who continued to receive RBC transfusions. In addition, this study showed a decreasing trend in LIC as well as normalisation of labile plasma iron levels. While the assessment of serum ferritin levels is an established, convenient and cost-effective laboratory technique for measuring body iron burden, quantifying redox-active forms of labile iron may provide additional insight into the clinical complications associated with iron overload (5, 28).

Previous clinical trials have demonstrated that deferasirox has a well-characterised and manageable safety profile in patients with MDS (17–19). The tolerability and safety of deferasirox in the eXtend and eXjange studies was comparable to that previously reported, with mild-to-moderate diarrhoea being the most common drug-related AE (17, 19, 27). Median serum creatinine increases were minimal.

The two studies reported here are associated with a number of limitations, primarily the small number of patients with MDS who were enrolled. In addition, because of the observational nature of the trials, monitors who could have controlled data collection were not employed; data collection was dependent on patients visiting their physicians regularly and doctors capturing the required information. Across the two studies, 31.1%, 41.3% and 28% of patients had missing International Prognostic Scoring System, karyotype and WHO classifications, respectively. It is therefore difficult to determine the effect of the severity of disease on the outcome of these patients. Furthermore, the proportion of patients with MDS who attended follow-up sessions over the course of the studies suggests that doctors’ and patients’ motivation diminished over time, as may be expected in observational trials. This may also reflect minor compliance or adherence problems in daily practice. Additionally, levels of serum ferritin may be influenced by acute and chronic inflammation, infections, collagen disease, malignancy and hepatic diseases (29). The observational nature of the eXtend and eXjange studies precluded the identification of comorbid clinical conditions that may have impacted serum ferritin analyses and therefore data should be interpreted cautiously (29).

In conclusion, previous deferasirox clinical trials have proven its efficacy in managing serum ferritin levels in iron-overloaded patients with transfusion-dependent MDS. Our naturalistic data extend findings from controlled clinical trials in demonstrating that deferasirox therapy is effective in managing iron overload in transfusion-dependent patients with MDS when used in a daily-routine situation in physicians’ medical practices.
